# Transient Elastography in the Diagnosis of Pediatric Non-alcoholic Fatty Liver Disease and Its Subtypes

**DOI:** 10.3389/fped.2022.808997

**Published:** 2022-03-30

**Authors:** Lin Yang, Yafei Zhu, Lu Zhou, Huimei Yin, Yan Lin, Guangsheng Wu

**Affiliations:** Department of Pediatrics, The Affiliated Hospital of Hangzhou Normal University, Hangzhou, China

**Keywords:** children with obesity, NAFLD, NASH, CAP, LSM

## Abstract

**Objective:**

To study the diagnosis efficacy of controlled attenuation parameters (CAP) and liver stiffness measurement (LSM) in the transient elastography of non-alcoholic fatty liver disease (NAFLD) and its subtypes in children with obesity.

**Methods:**

Retrospectively analyze children with obesity in the Childhood Obesity Clinic of the Affiliated Hospital of Hangzhou Normal University from July 2020 to March 2021. The correlation between clinical data and NAFLD subtypes was analyzed, and included the relevant clinical data into the receiver operating characteristic curve for diagnosis and prediction.

**Results:**

120 children aged between 6.1 and 17.8 years, with 70 males (58.33%), 50 females (41.67%), and a ratio of 1.4:1, were enrolled in the study. CAP and LSM correlated in all subtypes of NAFLD. The correlation was significant for diagnosing NAFLD in children with obesity when CAP > 258.00 dB/m and LSM > 4.65 kPa. It was also significant for NASH diagnosis when CAP > 276.00 dB/m and LSM > 5.15 kPa, while it was less significant for diagnosing NAFLD in children with obesity.

**Conclusions:**

CAP and LSM have diagnostic efficacy for NAFLD and its subtypes in children with obesity, with optimal predictive values of CAP > 258.00 dB/m and LSM > 4.65 kPa for NAFLD in children with obesity, and CAP > 276.00 dB/m and LSM > 5.15 kPa for NASH in children with obesity.

## Introduction

Childhood obesity has become a severe public health problem across the world. In China, childhood obesity is increasing at an alarming rate, far above the global average level (1). Obesity is one of the independent risk factors for NAFLD. While clinicians have extensively researched adult obesity, little research has been performed on the effects of childhood obesity on NAFLD. According to the liver histopathological features, NAFLD can be divided into non-alcoholic fatty liver (NAFL), non-alcoholic steatohepatitis (NASH), and associated liver fibrosis and cirrhosis. Liver biopsies are restricted for children due to their invasiveness and economic factors. It is crucial to adopt non-invasive methods to assist in diagnosing NAFL and NASH to halt the progression of liver fibrosis as early as possible. The main clinical screening method for pediatric NAFLD is the B-ultrasonography (B-scan) combined with a serum alanine transferase (ALT) test. However, due to the poor sensitivity and specificity of the B-scan in the assessment of NAFLD, increasing studies have pointed out the feasibility and effectiveness of transient elastography by FibroScan® in pediatric NAFLD diagnosis (2–5), as a useful supplement to traditional B-ultrasound. Transient elastography of the liver is a dynamic shear wave elastic imaging technology. The principle is to detect the propagation velocity of shear waves in liver tissue, to obtain the elastic modulus of liver tissue, namely liver stiffness measurements (LSM). At the same time, the ultrasonic wave is used to propagate attenuation in adipose tissue, and controlled attenuation parameters are quantitatively obtained according to the liver steatosis degree, namely controlled attenuation parameters (CAP). In clinical liver pathology studies, CAP and LSM have good stability and accuracy in treating liver steatosis and liver fibrosis (6, 7). This study mainly aimed at studying the significance of CAP and LSM in transient elastography to diagnose NAFLD and its subtypes.

## Materials and Methods

### Subjects

Children under the age of 18 were selected at the Childhood Obesity Clinic of the Affiliated Hospital of Hangzhou Normal University from July 2020 to March 2021. All the patients met the 2004 Body Mass Index Reference Norm for Screening Overweight and Obesity in Chinese Children and Adolescents set by the Working Group on Obesity in China (8). Secondary fatty liver diseases caused by thyroid disease, Wilson's disease, glycogen accumulation, and other diseases were excluded. All the enrolled subjects were simply obese.

### Methods

#### Physical Examination

The weight and height of the children were measured. Body mass index (BMI) was calculated by the following formula: BMI (kg/m^2^) = body mass (Kg)/height^2^(m^2^).

#### Blood Samples

Venous blood samples (3 ml each) were collected in the early morning after 12 h of fasting. Blood tests including 25-hydroxyvitamin D (25-D), Blood glucose (BG), alanine aminotransferase (ALT), aspartate aminotransferase (AST), gamma-glutamyl transferase total (GGT), cholesterol (TC), triglycerides (TG), low-density lipoprotein (LDL), insulin (INS), and uric acid (UA).

#### Transient Elastography by FibroScan®

CAP and LSM were measured by a special algorithm on the FibroScan502 Touch (Echosens, France) ultrasonic testing device, using a 3.5-MHz “M” or “XL” probe. Measurements were performed before breakfast or at least 2 h after a meal. In supine position and quiet state, the patients were tested 10 consecutive times from the right axillary front line to the axillary midline 7–8 intercostal. The median value was taken as the final result. The testing was completed by trained nursing staff.

#### Diagnostic Criteria

According to the Chinese Expert Consensus on the Diagnosis of Pediatric NAFLD (9), patients who met the following (1)–(5) items plus either item (6) or (7) were diagnosed with NAFLD: (1) Under the age of 18, with no history of drinking or alcohol consumption <140 g per week for males and 70 g per week for females; (2) With no other specific causes of fatty liver disease, including autoimmune liver disease, hypothalamic-pituitary disease, inflammatory bowel disease, thyroid disease, viral hepatitis, Wilson's disease, and Turner syndrome, and no pharmacochemical factors which may induce to fatty liver disease. (3) Some patients might have shown fatigue, dyspepsia, liver pain, hepatosplenomegaly, and other non-specific symptoms and body signs in addition to the clinical manifestations of the primary diseases. (4) Might have shown overweight, obesity (concentric obesity), elevated fasting blood glucose, lipid metabolism disorder, hypertension, and other metabolic syndromes. (5) Alanine transaminase (ALT) level higher than 1.5 times of the upper limit of the reference range (60 U/L) and lasting for more than 3 months. (6) Liver imaging manifestations met the diagnostic criteria of diffuse fatty liver. (7) Histological changes of liver biopsy met the pathological diagnostic criteria of fatty liver disease.

Patients who met items (1) and (2) plus either item (3) or (4) can be diagnosed with NAFL: (1) Met the clinical NAFLD diagnostic items (1)–(3). (2) Normal in the biochemical examination. (3) Liver imaging manifestations met the diagnostic criteria of diffuse fatty liver. (4) Histological changes of liver biopsy met the pathological diagnostic criteria of fatty liver disease.

NASH was diagnosed with items (1) + (2) + (3) or (1) + (4). (1) Meet the clinical NAFLD diagnostic items (1)–(3). (2) Unexplained serum ALT > 60 U/L and lasting for more than 3 months. (3) Liver imaging manifestations met the diagnostic criteria of diffuse fatty liver. (4) Histological changes of liver biopsy met the pathological diagnostic criteria of fatty hepatitis.

### Statistical Analysis

SPSS26.0 statistical software was used for all statistical analyses. Continuous variables are expressed as mean ± standard deviation (χ ± s), and classified variables are expressed by percentage ratio (%) represents. Pearson correlation analysis was used to describe the correlation between clinical variables and clinical subtypes of NAFLD. Pearson correlation coefficient (Cor) was used to express the correlation between variables. GraphPad Prism 8.0 software was used for plotting the receiver operating characteristic (ROC) curves to investigate the diagnostic efficacy of CAP and LSM for NAFLD, NAFL, and NASH. The optimal diagnostic threshold point on the ROC curve was selected by the Youden index. *P* < 0.05 was established as the significance level.

## Results

### Clinical Features

One hundred and twenty children, among which 70 male (58.33%) and 50 female (41.67%), aged from 6.1 to 17.8 years(10.85 ± 2.33)with obesity, were enrolled in this study. The gender ratio was 1.4:1. There were 61 NAFLD diagnosed cases, accounting for 50.83% of all the children with obesity, of which 45 were male (73.77%), and 16 were female (26.33%). There were 44 NAFL diagnosed cases, accounting for 72.13% of all the NAFLD children, among which 32 were male (72.73%), and 12 were females (19.67%). There were 17 cases diagnosed with NASH, accounting for 27.87% of all the NAFLD children, among which 14 were male (82.35%), and three were females (17.65%). No liver biopsy was performed in the 120 cases. Clinical data were included in Pearson correlation analysis, and the results are shown in Table 1. CAP and LSM were correlated in all subtypes of NAFLD, *P* < 0.005.

**Table 1:** Analysis of correlation between clinical data and non-NAFLD, NAFLD, NAFL, NASH.

**Clinical data**		**Non-NAFLD** **(*n* = 59)**	**NAFLD** **(*n* = 61)**	**NAFL** **(*n* = 44)**	**NASH** **(*n* = 17)**
Age	$\bar{\chi }$ ± s	9.86 ± 2.07	11.79 ± 2.18	11.39 ± 2.04	12.82 ± 2.24
	Cor	−0.415	0.415	0.179	0.348
	*P*	<0.001	<0.001	0.051	<0.001
Gender	M/F	25/34	45/16	33/11	12/5
	Cor	−0.318	0.318	0.257	0.101
	*P*	<0.001	<0.001	0.005	0.272
BMI (kg/m^2^)	$\bar{\chi }$ ± s	23.43 ± 2.75	27.49 ± 4.30	27.66 ± 4.60	27.05 ± 3.48
	Cor	−0.492	0.492	0.400	0.154
	*P*	<0.001	<0.001	<0.001	0.093
CAP (dB/m)	$\bar{\chi }$ ± s	242.05 ± 40.59	281.16 ± 41.71	277.73 ± 43.62	290.06 ± 35.95
	Cor	−0.432	0.432	0.266	0.252
	*P*	<0.001	<0.001	0.003	0.005
LSM (kPa)	$\bar{\chi }$ ± s	4.31 ± 1.46	5.59 ± 1.51	5.43 ± 1.28	6.02 ± 1.95
	Cor	−0.400	0.400	0.221	0.269
	*P*	<0.001	<0.001	0.016	0.003
25-D (nmol/L)	$\bar{\chi }$ ± s	29.64 ± 11.29	45.70 ± 14.29	47.57 ± 13.55	40.80 ± 15.47
	Cor	0.153	−0.153	−0.005	−0.213
	*P*	0.104	0.104	0.955	0.022
BG (mmol/L)	$\bar{\chi }$ ± s	5.22 ± 0.27	5.21 ± 0.31	5.25 ± 0.33	5.11 ± 0.24
	Cor	0.021	−0.021	0.082	−0.142
	*P*	0.819	0.819	0.380	0.125
ALT (U/L)	$\bar{\chi }$ ± s	20.76 ± 13.56	41.97 ± 30.98	27.34 ± 11.77	79.82 ± 33.39
	Cor	−0.406	0.406	−0.122	−0.752
	*P*	<0.001	<0.001	0.183	<0.001
AST (U/L)	$\bar{\chi }$ ± s	24.76 ± 11.05	29.10 ± 12.28	22.95 ± 4.63	45.00 ± 14.64
	Cor	−0.177	0.177	−0.250	0.599
	*P*	0.053	0.053	0.006	<0.001
GGT (U/L)	$\bar{\chi }$ ± s	18.75 ± 15.47	25.69 ± 12.11	24.41 ± 12.55	29.00 ± 10.50
	Cor	−0.245	0.245	0.115	0.193
	*P*	0.007	0.007	0.213	0.035
TC (mmol/L)	$\bar{\chi }$ ± s	4.21 ± 0.72	4.17 ± 0.86	4.18 ± 0.83	4.16 ± 0.97
	Cor	0.021	−0.021	−0.009	−0.017
	*P*	0.830	0.830	0.928	0.859
TG (mmol/L)	$\bar{\chi }$ ± s	1.08 ± 0.54	1.54 ± 1.63	1.48 ± 1.75	1.70 ± 1.29
	Cor	−0.183	0.183	0.097	0.123
	*P*	0.060	0.060	0.322	0.208
LDL (mmol/L)	$\bar{\chi }$ ± s	2.44 ± 0.54	2.43 ± 0.61	2.42 ± 0.61	2.45 ± 0.64
	Cor	0.011	−0.011	−0.018	0.010
	*P*	0.912	0.912	0.851	0.918
UA (h mol/L)	$\bar{\chi }$ ± s	367.88 ± 82.54	434.03 ± 94.00	442.17 ± 96.68	412.69 ± 85.77
	Cor	−0.351	0.351	0.329	0.042
	*P*	<0.001	<0.001	<0.001	0.662
INS (pmol/L)	$\bar{\chi }$ ± s	80.46 ± 40.80	125.67 ± 65.99	125.64 ± 73.57	125.76 ± 45.22
	Cor	−0.379	0.379	0.274	0.155
	*P*	<0.001	<0.001	0.004	0.112

**Figure 1:**
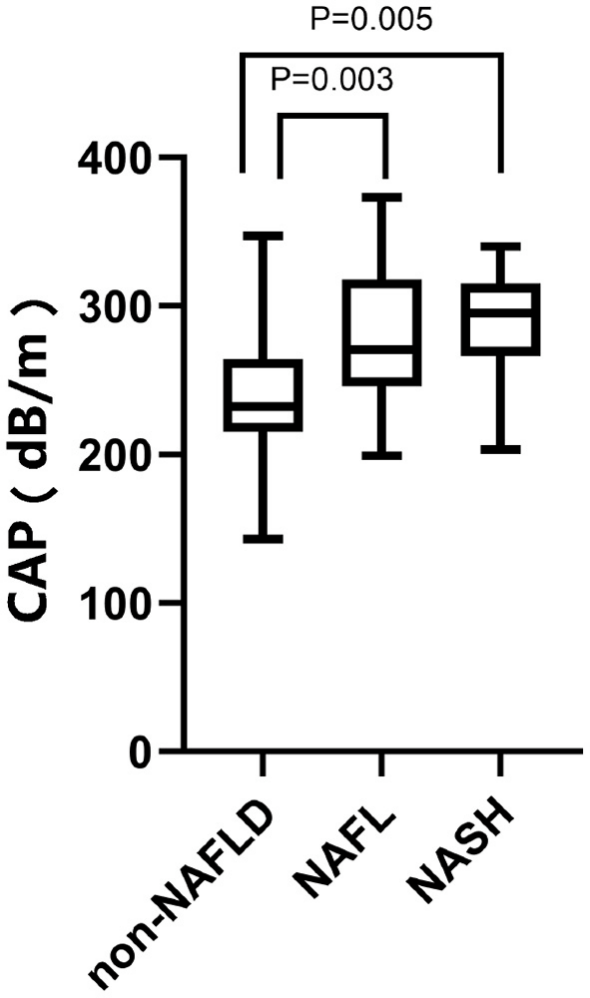
CAP values of 120 children with obesity. From left to right: 242.05 ± 40.586 dB/m in the non-NAFLD group, *P* < 0.0001; 277.73 ± 43.620 dB/m in the NAFL group, *P* = 0.003; 290.06 ± 35.949 dB/min the NASH group, *P* = 0.005.

**Figure 2:**
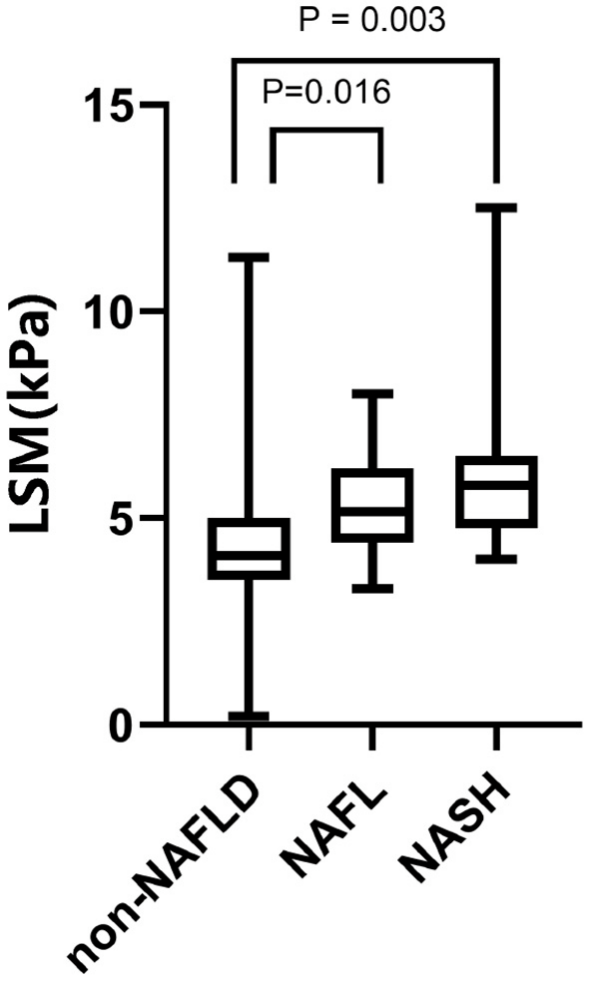
LSM values of 120 children with obesity. From left to right: 4.307 ± 1.459 kPa in the NAFLD group, *P* < 0.0001, 5.425 ± 1.28 kPa in the NAFL group, *P* = 0.016; 6.024 ± 1.954 kPa in the NASH group, *P* = 0.003.

Assessment of the diagnostic efficacy of CAP and LSM for NAFLD, NAFL, and NASH in children with obesity. The maximum Youden Index was used to determine the optimal threshold values. The results are shown in Figures 1, 2.

**Figure 3:**
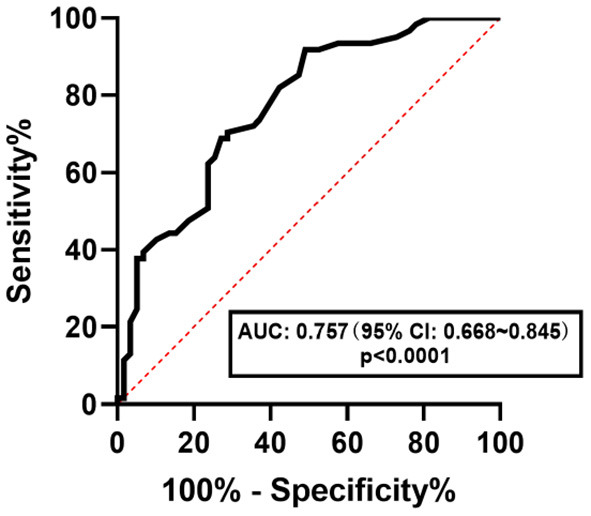
ROC curve of CAP in predicting NAFLD in children with obesity.

**Figure 4:**
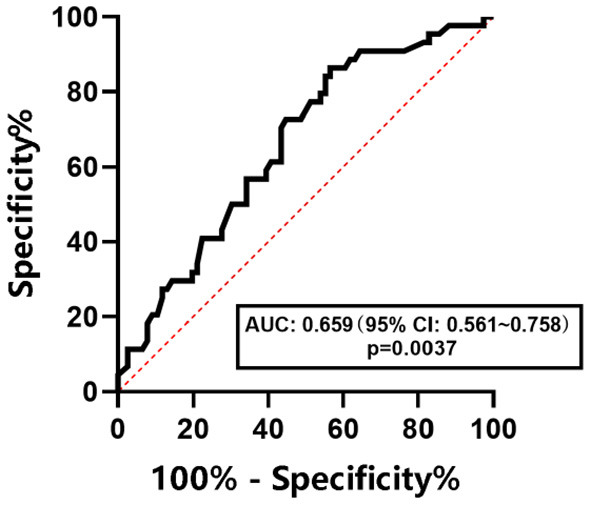
ROC curve of CAP in predicting NAFL in children with obesity.

**Figure 5:**
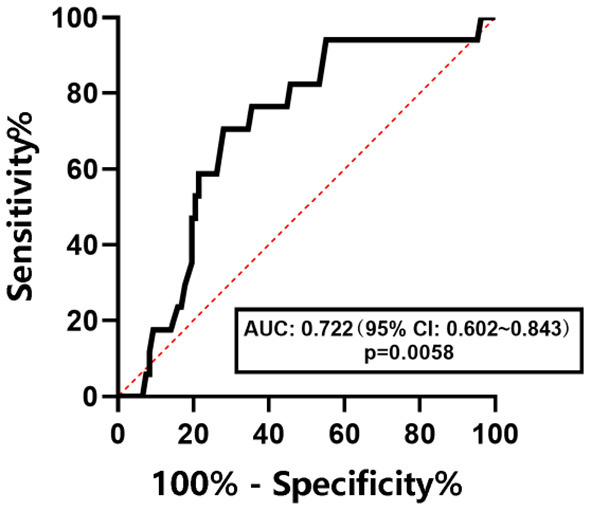
ROC curve of CAP in predicting NASH in children with obesity.

The ROC curve was used to determine the diagnostic efficacy of CAP to NAFLD, NAFL, and NASH in children with obesity. The results are shown in Figures 3–5. The optimal predictive value of CAP for NAFLD in children with obesity was 258.00 dB/m (AUC: 0.757; 95% CI: 0.668 ~ 0.845, *p* < 0.0001), with a sensitivity of 67.2%and a specificity of 67.2%. The optimal predictive value of CAP for NAFL in children with obesity was 262.50 dB/m (AUC: 0.659; 95% CI: 0.561 ~ 0.758, *p* = 0.0037), with a sensitivity of 59.1% and a specificity of 60.5%.The optimal predicted value of CAP for NASH in children with obesity was 276.00 dB/m (AUC: 0.722; 95% CI: 0.602 ~ 0.843, *p* = 0.0058), with a sensitivity of 70.6% and a specificity of 72.8%.

**Figure 6:**
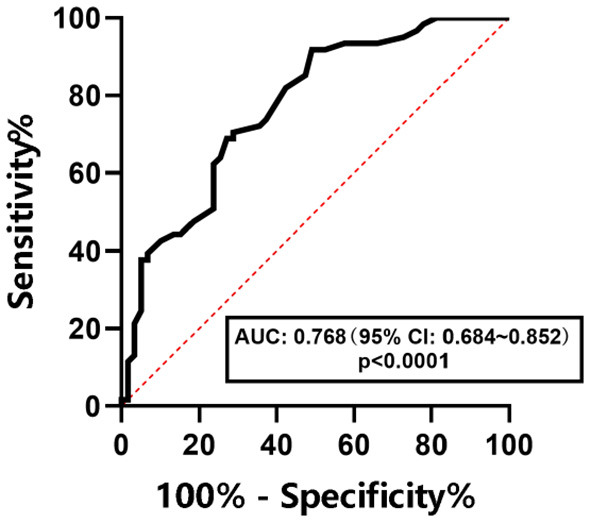
ROC curve of LSM in predicting NAFLD in children with obesity.

**Figure 7:**
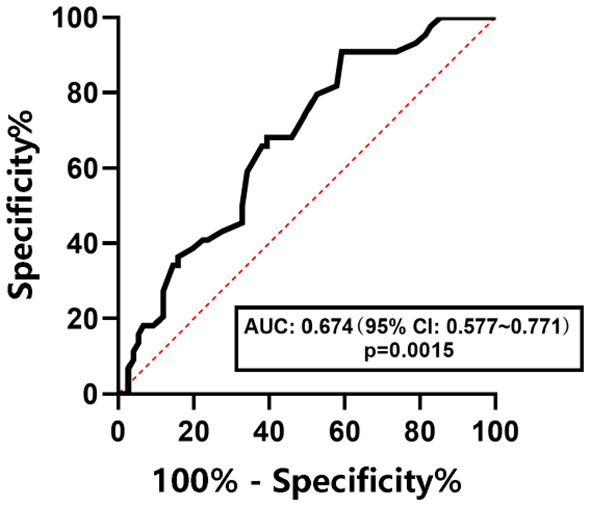
ROC curve of LSM in predicting NAFL in children with obesity.

**Figure 8:**
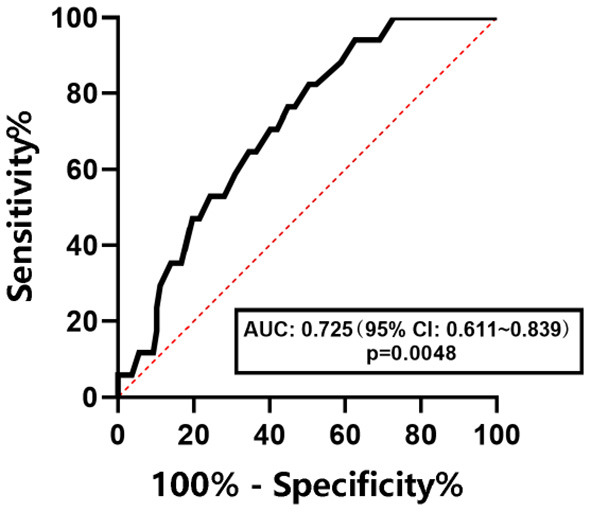
ROC curve of LSM in predicting NASH in children with obesity.

The ROC curves were used to determine the diagnostic efficacy of LSM to NAFLD, NAFL, and NASH in children with obesity. The results are shown in Figures 6–8. The optimal predictive value of LSM for NAFLD in children with obesity was 4.65 kPa (AUC: 0.768; 95% CI: 0.684 ~ 0.852, *p* < 0.0001), with a sensitivity of 70.5% and a specificity of 70.7%.The optimal predictive value of LSM for NAFL in children with obesity was 4.95 kPa (AUC: 0.674; 95% CI: 0.577 ~ 0.771, *p* = 0.0015), with a sensitivity of 61.4% and a specificity of 64.5%.The optimal predictive value of LSM for NASH in children with obesity was 5.15 kPa (AUC: 0.725; 95% CI: 0.611 ~ 0.839, *p* = 0.0048), with a sensitivity of 64.7% and a specificity of 65.0%.

## Discussion

The obesity rate of Chinese children is increasing yearly with a trend toward lower age (10). The target organs' damage from obesity, especially the liver, is growing at an alarming rate. In China, the ALT value threshold in children for pediatric NAFLD diagnosis follows the standard for adults. However, increased scholars at home and abroad have suggested that children's ALT threshold in screening NAFLD should be lower than adults'. Lu et al. (11) revised the serum ULN-ALT (25 U/L for males and 20 U/L for females), showing that the prevalence of NASH in children in China may be underestimated. NASH is a subtype of NAFLD that can develop into cirrhosis, hepatocellular carcinoma, and even death (12), and children usually suffer more severe NAFLD than adults (13), among all about 15%were accompanied with NASH and associated cirrhosis when diagnosed with NAFLD (14, 15). In this study, there were more male than female children with obesity. The same gender difference was observed in the NAFLD cases, consistent with the assumption from most researchers about the protective effect of estrogen. Moreover, adults studies showed that the male gender was one of the risk factors of NASH (2, 16, 17), but this study indicated that males were dominant in both NAFLD and its subtypes. However, more studies are needed to confirm the relevance of gender and the prevalence of NAFLD since there is a lack of large-sample clinical research of pediatric NAFLD at home and abroad.

The clinical application and research of transient elastography in adult NAFLD have been relatively mature. It is of great significance in adult NAFLD and can be used to recognize fatty liver stages and diagnose severe liver fibrosis (7, 18, 19). However, most pediatricians are still following the adult reference values for children, which cannot rule out misdiagnose and omissions.

In our study, transient elastography by FibroScan, a non-invasive method, was used for assessing children with obesity. Since liver biopsy was not performed, the direct relationship between transient elastography of the liver and the degree of liver steatosis and fibrosis cannot be directly established. However, CAP and LSM have high accuracy in diagnosis with conventional ultrasound combined with ALT currently applied in clinical practice. Nevertheless, it is worth noting that CAP and LSM have higher diagnostic efficacy in NAFLD and NASH than in NAFL.

Of all the clinic researches in children, Kwon et al. (2) reported a specific value range for CAP and LSM in children with obesity between the ages of 5 and 15, 244.4–340.98 dB/m for CAP and 3.85–7.77 kPa for LSM. However, the study did not provide a diagnostic threshold value. An American study by Ferraioli et al. (18) in 2017 suggested that CAP is advantageous for assessing liver steatosis in children. About 9.3% of the children with normal US results were detected with liver steatosis through CAP; thus, they recommended CAP = 249 bB/m as a threshold value for liver steatosis diagnosis. De Paor et al. (20) examined the CAP values of different histological steatosis grades, confirmed its significance for steatosis detection, and suggested using CAP = 225 bB/m as a possible optimal threshold for steatosis detection in children. However, the small sample size of this study limited its reliability, and the mean time interval between liver biopsy and CAP testing was 1.3 months. Therefore, the error due to rapid liver steatosis cannot be ruled out. Runge et al. (21) used the magnetic resonance spectroscopy proton density fat fraction (MRS-PDFF) as a reference standard to compare CAP and traditional ultrasound differences in liver steatosis of children with obesity. It was proposed that CAP had higher accuracy with an optimal threshold value of 277 dB/m. In our study, the optimal CAP threshold value for NAFLD was 258.00 dB/m, different from the mentioned studies. This may be because, in our study, patients with mild hepatic steatosis were not detected by B-ultrasound, which is also a limitation of our study. Another reason may be due to differences samples size and race since all the children in this study were from China without a control group of non-obese children. Furthermore, most children were diagnosed with fatty liver by ultrasound rather than liver biopsy, and PDFF was only performed on a few children to detect liver fat content. All the above aspects are the limitations of our study.

Our study evaluated the diagnostic efficacy of CAP and LSM to the occurrence of NAFLD, NAFL, and NASH in children with obesity. Although they all showed diagnostic efficacy, there were differences among the three. The diagnostic efficacy, the sensitivity, and the specificity of the optimal predictive values of CAP and LSM to NAFLD and NASH were higher than those of the NAFL group. The CAP and LSM optimal predictive for NAFLD in children with obesity are 258.00 dB/m and 4.65 kPa, and the sensitivity and specificity of the LSM optimal predictive value were better than those of CAP. The CAP and LSM diagnostic efficacy to NASH in children with obesity were not far from the previous group. The optimal predictive values of CAP and LSM for NASH in children with obesity were 276.00 dB/m and 5.15 kPa. The sensitivity and the specificity of the optimal predictive value of CAP were better than LSM. In the NAFL group, there was little diagnostic efficacy of CAP and LSM, poor sensitivity, and poor specificity of their optimal predictive values. The diagnostic efficacy of CAP and LSM was confirmed, but there may be different advantages between them in NAFLD and its subtypes. Therefore, a larger-sample clinical study is required to confirm it.

In conclusion, with NAFLD cases gradually increasing in children and liver biopsy showing obvious limitations, transient elastography has a distinct advantage as a method because of its non-invasiveness, economic value, and ease of use in implementation at the clinic. This study confirmed its diagnostic efficacy for NAFLD and its subtypes in children with obesity. Moreover, CAP > 258.00 dB/m and LSM > 4.65 kPa can serve as NAFLD diagnostic reference values in children with obesity, and CAP > 276.00 dB/m and LSM > 5.15 kPa as the NASH diagnostic reference values in children with obesity.

## Data Availability Statement

The original contributions presented in the study are included in the article/supplementary material, further inquiries can be directed to the corresponding author.

## Ethics Statement

This study was approved by the Medical Ethics Committee, the Affiliated Hospital of Hangzhou Normal University [approval number: 2021-(E2)-HS-061]. Written informed consent to participate in this study was provided by the participants' legal guardian/next of kin.

## Author Contributions

YZ, LZ, HY, and YL: data collection. LY: data sorting and manuscript writing. GW: manuscript revision and project supervision. All authors contributed to the article and approved the submitted version.

## Funding

This study was supported by Hangzhou Medical Science and Technology Project (A20210060).

## Conflict of Interest

The authors declare that the research was conducted in the absence of any commercial or financial relationships that could be construed as a potential conflict of interest.

## Publisher's Note

All claims expressed in this article are solely those of the authors and do not necessarily represent those of their affiliated organizations, or those of the publisher, the editors and the reviewers. Any product that may be evaluated in this article, or claim that may be made by its manufacturer, is not guaranteed or endorsed by the publisher.
